# Inoculum microbial mass is negatively related to microbial yield and positively to methane yield *in vitro*

**DOI:** 10.1017/jns.2024.37

**Published:** 2024-09-20

**Authors:** Xiaoyu Zhang, Fenja Klevenhusen, Angela Sünder, Marcus Clauss, Jürgen Hummel

**Affiliations:** 1 Environmentally Sustainable Animal Nutrition, Faculty of Organic Agricultural Sciences, University of Kassel, Witzenhausen, Germany; 2 Animal Nutrition Physiology, Department of Animal Sciences, University of Göttingen, Göttingen, Germany; 3 Clinic for Zoo Animals, Exotic Pets and Wildlife, Vetsuisse Faculty, University of Zurich, Zürich, Switzerland; 4 Ruminant Nutrition, Department of Animal Sciences, University of Göttingen, Göttingen, Germany

**Keywords:** Degraded substrate partitioning, Initial microbial mass, Metabolic hydrogen, Methane, Stoichiometric relationship

## Abstract

Ruminal microbes catabolise feed carbohydrates mainly into SCFA, methane (CH_4_), and carbon dioxide (CO_2_), with predictable relationships between fermentation end products and net microbial increase. We used a closed *in vitro* batch culture system, incubating grass and maize silages, and measured total gas production at 8 and 24 h, as well as the truly degraded substrate, the net production of SCFA, CH_4_, and microbial biomass at 24 h, and investigated the impact of silage type and inoculum microbial mass on fermentation direction. Net microbial yield was negatively correlated with total gas at 8 h (P < 0•001), but not at 24 h (P = 0•052), and negatively correlated with CH_4_ production (P < 0•001). Higher initial inoculum microbial mass was related to a lower net microbial yield (P < 0•001) but a higher CH_4_ production (P < 0•001). A significant difference between grass silage and maize silage was detected within the context of these relationships (P < 0•050). The metabolic hydrogen (2H) recovery was 102.8 ± 12.3 % for grass silages and 118.8 ± 13.3% for maize silages. Overall, grass silages favoured more substrate conversion to microbial biomass and less to fermentation end products than maize silage. Lower inoculum microbial mass facilitated more microbial growth and, because of the 2H sink by microbial synthesis, decreased CH_4_ production.

## Introduction

The ruminal microbiome breaks down plant carbohydrates primarily into SCFA, methane (CH_4_), and carbon dioxide (CO_2_). During the breakdown of the substrates, electrons are released and bound in different metabolic pathways, transferred by redox cofactors such as NAD^+^/NADH and NADP^+^/NADPH. These cofactors transfer two electrons and hydrogen atoms; therefore, it is suggested that metabolic hydrogen (2H) could be used as a unifying principle to study rumen fermentation as a whole.^(1,2)^ Based on previous research, it is indicated that the production of one mole of acetate and one mole of butyrate produces two moles of 2H each, while the production of one mole of propionate consumes one mole of 2H; further, the production of one mole CH_4_ consumes four moles of 2H.^(3–5)^ Nevertheless, other factors could potentially influence the electron flow, such as unsaturated fatty acids and nitrate, dihydrogen gas, or valerate.^(5)^ However, under natural feeding situations, these factors are of minute quantity. Because the produced 2H needs to be consumed within the ruminal fermentation system, a tight relationship among fermentation end products exists.^(6,7)^

However, another metabolic pathway might have a significant impact on ruminal fermentation balance and electron flow and was relatively rarely mentioned in existing studies — the synthesis of microbial biomass; this process will consume 2H (e.g. to produce fatty acids for microbial cell membranes) and, therefore, reduces CH_4_ emission.^(1,8)^ Blümmel *et al.* ^(9)^ reported a negative relationship between the production of microbial mass and gas production per unit of degraded substrate based on the Hohenheim gas test (HGT). The same study also defined the partitioning factor (PF) as the ratio of truly degraded organic matter (OM) to total gas production; a higher PF indicates more degraded substrate is partitioned to microbial synthesis and less to fermentation end products. The negative relationship between microbial mass and gas production might stem from CH_4_ due to competition for 2H. However, quantifying the potential of 2H consumption by microbial synthesis is challenging; different magnitudes were estimated by different authors, which were mainly based on assumptions of microbial composition and stoichiometric calculations.^(10–12)^

The relationship between SCFA and CH_4_, as well as the potential 2H consumption by microbial synthesis, is challenging to verify *in vivo* due to difficulties in measuring accumulated amounts during fermentation. Therefore, we used the closed *in vitro* system HGT modified by Blümmel *et al.* ^(9)^ for the incubation of grass and maize silages to measure the net production of SCFA, CH_4_, and microbial mass. Across six different experimental runs, we observed variations in the initial inoculum microbial mass; this was likely caused by unintended variation in the time between the last feeding or drinking event and the inoculum sampling or by inadvertent variation in the exact sampling site in the rumen. These variations emerged as influential factors in *in vitro* fermentation.

We assumed that 100% of 2H released during acetate and butyrate production is consumed by propionate and CH_4_ production (the impact of isobutyrate, valerate, and other metabolites on redox balance was omitted because of the assumed minute quantity), and therefore, the amount of CH_4_ could be calculated through the amount of acetate, propionate, and butyrate as CH_4_ = 0.5 × Acetate – 0.25 × Propionate + 0.5 × Butyrate (simply derived based on the previously mentioned relationship; unit: mol^(3,7)^). The calculated CH_4_ production will be compared to the actually measured amounts. A negative relationship between microbial and CH_4_ yield was expected, and PF was expected to be a useful indicator of the metabolic state of the *in vitro* microbiome, that is, whether the microbiome was more in a growth or a maintenance stage. By doing these, we aimed to acquire novel insights into ruminal fermentation balance and electron flow and the effects of the initial microbial mass and feed types as influencing factors.

## Materials and methods

### Hohenheim gas test (HGT)

Fifteen grass silage samples and eight maize silage samples were collected from ten commercial dairy farms in the northwest of Germany, dried to constant weight at 60°C (Memmert ULE 500, Memmert GmbH + Co.KG, Schwabach, Germany), and incubated in HGT syringes for 24 h (each sample in duplicate). The dry matter (DM) was determined by drying subsamples, at 103°C to constant weight (Memmert ULE 500, Memmert GmbH + Co.KG, Schwabach, Germany). Rumen fluid was collected from two rumen-fistulated Holstein-Friesian cows before the morning feeding (cows were fed 9 kg mixed grass hay, 600 g compound feed, and 75 g mineral supplement per animal and day), filtered through two layers of cheesecloth, placed in a prewarmed CO_2_-filled container, transferred to the laboratory, and then mixed with the medium described by Menke *et al.* ^(13)^ The donor animal care and experimental procedures were conducted according to the German Guidelines and Regulations on Animal Care (Deutsches Tierschutzgesetz) (LAVES-Aktenzeichen 33.9-42502-05-18A269). The dried silage samples (about 200 mg DM) were ground through 1 mm sieve (cutting mill SM300; Retsch Ltd., Haan, Germany) and put into glass syringes and mixed with 30 ml inoculum. Three blank syringes without substrates were also injected with 30 ml of inoculum to calibrate the gas production. The gas volume produced was recorded at 8 and 24 h after initiation. After 24 h, the incubation was terminated by placing the syringes on ice.

### Measurements

The nutrient composition of silage samples was analysed according to the standard methods of the Association of German Agricultural Analysis and Research Institutes^(14)^ for DM, OM, crude ash, crude protein (CP; N × 6.25), ether extract (EE), neutral detergent fibre (NDF), acid detergent fibre (ADF), and acid detergent lignin (ADL) (Table 1). The neutral detergent fibre was analysed after adding amylase, and NDF, ADF, and ADL were corrected for residual ash.

**Table 1.** The feed nutrient composition, metabolisable energy, true and apparent degradability (fifteen grass silages and eight maize silages samples)

		OM	CP	EE	NDF_om_	ADF_om_	ADL_om_	ME	AOMD	TOMD
		g/kg DM						MJ/kg DM	%	
Grass silage (*n* = 15)	Mean	891.7	173.8	42.5	520.1	319.1	33.0	9.9	42.7	80.8
	SD	27.2	29.70	6.04	48.04	35.67	13.58	0.84	6.97	6.74
Maize silage (*n* = 8)	Mean	966.7	73.6	28.8	408.2	227.2	25.0	10.8	43.2	75.4
	SD	3.2	7.17	3.89	23.09	10.55	3.70	0.33	3.65	2.34

DM, dry matter; OM, organic matter; CP, crude protein; EE, ether extract; NDF_om_, neutral detergent fibre corrected for residual ash; ADF_om_, acid detergent fibre corrected for residual ash; ADL_om_, acid detergent lignin corrected for residual ash; ME, metabolisable energy; AOMD, apparent organic matter degradability; TOMD, true organic matter degradability; SD, standard deviation.

The gas sample after 24 h was collected from one of the duplicate HGT syringes; 15 ml of gas sample were collected and injected into the 5.9 ml vacuum tube (Exetainer® 5.9 ml Evacuated Flat Bottom Vial; Labco Limited, Lampeter, United Kingdom) for gas composition (H_2_, O_2_, N_2_, CH_4_, CO_2_) measurement by GC (TRACE™ 1300 Series GC, Thermo Scientific, Waltham, MA, USA) equipped with a thermal conductivity detector. The used column was a 30 m × 0.53 mm in size TracePLOT TG-BOND Msieve 5A (Thermo Scientific, Waltham, MA, USA), and argon was used as a carrier gas.

The *in vitro* apparently and truly degraded substrate was measured; the procedure of Blümmel *et al.* ^(9)^ was modified, collecting the entire content after incubation from one of the duplicate HGT syringes, gently injecting it into a 50 ml plastic centrifuge tube (Carl Roth GmbH + Co. KG, Karlsruhe, Germany). The tube was centrifuged at 20000 g for 30 min at 4°C, and the supernatant fraction was sampled for SCFA measurement. The remaining supernatant was carefully discarded to minimise any loss of the solid fraction. The former HGT syringe was then rinsed with totally 40 ml NaCl (4 g/l) two to four times, ensuring no particles attached to the glass surface. All the rinse liquid was introduced to the centrifuge tube. The tube was again centrifuged at 20000 g for 30 min at 4°C, and the supernatant fraction was carefully discarded. Subsequently, the tube was dried at 60°C for 2 d, followed by drying at 103°C overnight. Organic matter was determined afterwards via muffle furnace combustion at 550°C for 4 h. The apparently degraded OM was calculated as the difference between the initial OM before incubation and the collected OM after incubation.

The truly degraded OM was calculated as the difference between the initial OM before incubation and the collected and boiled OM after incubation. Therefore, the entire content from the other duplicate HGT syringe was carefully transferred into a Gerhardt fibre bag (FibreBags ADF, C. Gerhardt GmbH & Co. KG, Königswinter, Germany). The HGT syringe was rinsed with a total of 80 ml NaCl (4 g/l) three times, to ensure no particles attached to the glass surface. All the rinsing liquid was introduced into the Gerhardt fibre bag. The bags were boiled with a neutral detergent solution (NDS) for 1 h to remove microbial mass and then dried at 103°C overnight. Organic matter content was determined via muffle furnace combustion at 550°C for 4 h.

**Fig. 1.**
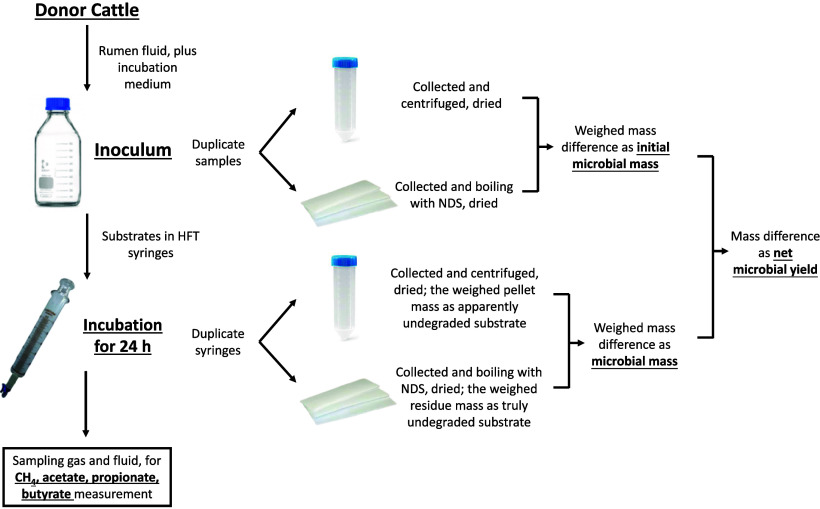
Determination of microbial mass and sampling of gas and fluid. Microbial mass is expected to be removed through boiling with a neutral detergent solution (NDS).

The microbial mass in OM was considered as the difference between the apparently and truly degraded OM (Fig. 1). For each of the six runs, the initial microbial mass before incubation was measured in the same way in the inoculum. Net microbial yield was calculated as the microbial mass at the end of fermentation minus the initial microbial mass (Fig. 1).

For analysis of SCFA, 800 µl of the supernatant was injected into an Eppendorf tube, filled with 150 µl 25% metaphosphoric acid and 50 µl ISTD (4% 2-methylvaleric acid in formic acid). The mixture was again centrifuged at 16600 g for 20 min at 10°C and analysed via GC (GC-17A, SHIMADZU, Kyoto, Japan) equipped with a flame ionisation detector. The Stabilwax w/Integra-Guard column (Restek Corporation, Bellefonte, United States) was 30 m × 0.25 mm in size, and nitrogen gas was used as a carrier gas.

### Calculation and Statistics

The PF after 24 h was calculated as suggested by Blümmel *et al.* ^(9)^ as:

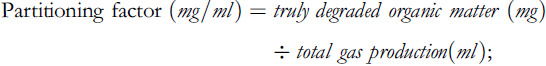

it serves as a measure of the variation between fermentation end products (gas and SCFA) and microbial mass per unit degraded substrate. A higher PF indicates more degraded substrate was directed towards microbial synthesis.

We did not measure the initial SCFA concentration in the inoculum; therefore, we estimated it by establishing a linear regression between true OM degradability as an independent variable and SCFA concentration as a dependent variable for grass silage samples (in which the fitting of this relationship was higher than in the maize silage samples); the intercept was assumed to be the initial SCFA concentration in the inoculum (Appendix). The reliability of this assumption is grounded in the reported linear relationship between SCFA and digestibility.^(15,16)^ The net SCFA concentration was calculated as the difference between the measured SCFA concentration after incubation and the estimated initial SCFA concentration in the inoculum.

The theoretical values of CH_4_ yield were calculated as^(3,7)^:

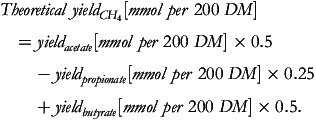




The 2H recovery was calculated as^(1,2)^:

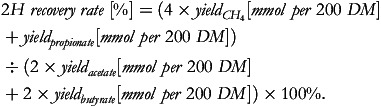




The truly degraded OM and the net microbial, SCFA, and gas yields were standardised on the base of 200 mg DM substrate.

The average values between grass and maize silages were compared with a one-way ANOVA. The relationships among variables were analysed using R (version 3.5.2) with a linear mixed model. Net microbial yield and silage type were used as fixed effects, and individual silage sample was used as a random effect with dependent variables of gas production in 8 h, gas production in 24 h, ratio between gas production in 8 and 24 h, CH_4_ production in 24 h, CO_2_ production in 24 h, and ratio between CH_4_ and CO_2_ production in 24 h as:

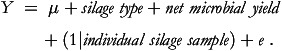




Initial microbial mass and silage type were used as fixed effects, and individual silage sample was used as a random effect with dependent variables of net microbial yield, CH_4_ production in 24 h, ratio between CH_4_ and CO_2_ production in 24 h, gas production in 8 h, and gas production in 24 h as:

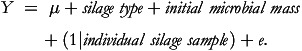




The μ represents the overall mean, and e represents the residual error. The model’s explanatory power was estimated using marginal R^2^ (R^2^_m_) and conditional R^2^ (R^2^_c_), where R^2^_m_ represents the variance explained by the fixed effects alone and R^2^_c_ represents the total variance explained by the model for both fixed and random effects. The number of independent observations was 144 (24 silage samples × 6 replicates); the significance level was set as 0•050.

## Results

Maize silages produced more gas than grass silages in 8 h and 24 h, but the difference was greater after 8 than after 24 h (P < 0•001; Table 2). However, the proportions of CH_4_ and CO_2_ after 24 h were similar between the two types of silage (P = 0•471). The true OM degradability of maize silages after 24 h was lower than of grass silages (P < 0•001; Table 1), and fermentation of maize silages produced more propionate, butyrate and CH_4_, and less microbial mass (P < 0•050; Table 2). The initial microbial mass ranged from 21.7 to 44.5 mg per 30 ml inoculum in six runs. The estimated initial SCFA concentration in the inoculum ranged from 19.8 to 28.3 µmol/ml for acetate, from 0.13 to 5.20 µmol/ml for propionate, and from 0 to 3.52 µmol/ml for butyrate in six runs.

**Table 2.** The fermentation parameters

		Grass silage (*n* = 15)	Maize silage (*n* = 8)	*P* value
*In 8 h*				
Gas volume	ml/200mg DM	13.1 ± 4.14	26.5 ± 3.05	<0.001
*In 24 h*				
Gas volume	ml/200mg DM	41.0 ± 5.63	50.1 ± 1.99	<0.001
CH_4_:CO_2_ ratio	ml/ml	0.22 ± 0.03	0.22 ± 0.02	0.471
Microbial yield	mg/200mg DM	35.6 ± 5.81	31.0 ± 4.77	<0.001
Partitioning factor	mg/ml	3.6 ± 0.33	2.9 ± 0.10	<0.001
Acetate production	mmol/200mg DM	0.67 ± 0.11	0.67 ± 0.09	0.867
Propionate production		0.41 ± 0.07	0.44 ± 0.05	0.012
Butyrate production		0.13 ± 0.04	0.16 ± 0.04	<0.001
CH_4_ production		0.31 ± 0.06	0.38 ± 0.04	<0.001
2H recovery^a^	%	102.8 ± 12.28	118.8 ± 13.29	<0.001

*Note:* Values are arithmetic means ± standard deviation and presented as net production during 24 h of fermentation.

a 2H recovery was calculated as (4 × CH_4_ + Propionate)/(2 × Acetate + Butyrate) × 100%.

**Fig. 2.**
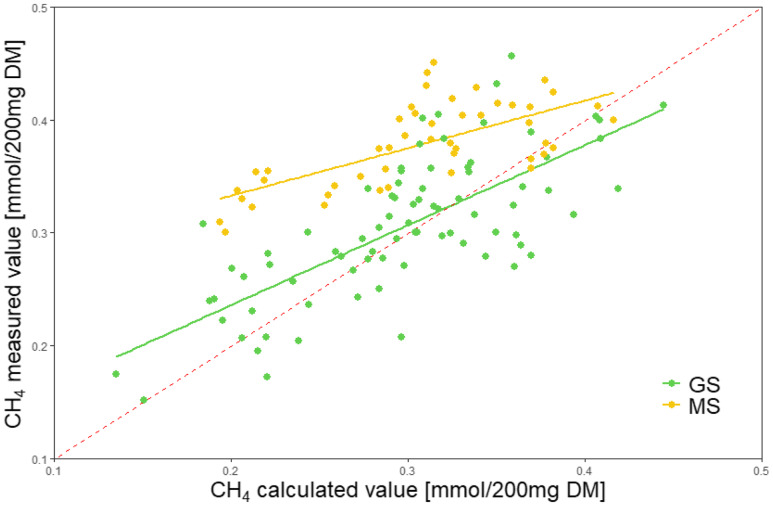
Comparison of measured and theoretical values of CH_4_ production. The dashed line represents when measured and theoretical values are equal. GS, grass silage; MS: maize silage. The linear regression equation between measured (Y) and calculated (X) CH_4_ is Y = 0.09 + 0.71 × X (R^2^ = 0.53) for grass silage and Y = 0.25 + 0.42 × X (R^2^ = 0.46) for maize silage.

The 2H recovery calculated through SCFA and CH_4_ yields were 102.8% and 118.8% for grass and maize silages, respectively (Table 2). As shown in Fig. 2, the linear regression equation between measured (Y) and calculated (X) CH_4_ is Y = 0.09 + 0.71 × X (R^2^ = 0.53; 95% CI 0•05, 0•14 for the intercept, and 0•56, 0•86 for the slope) for grass silages and Y = 0.25 + 0.42 × X (R^2^ = 0.46; 95% CI 0•21, 0•29 for the intercept and 0•28, 0•56 for the slope) for maize silages.

**Fig. 3.**
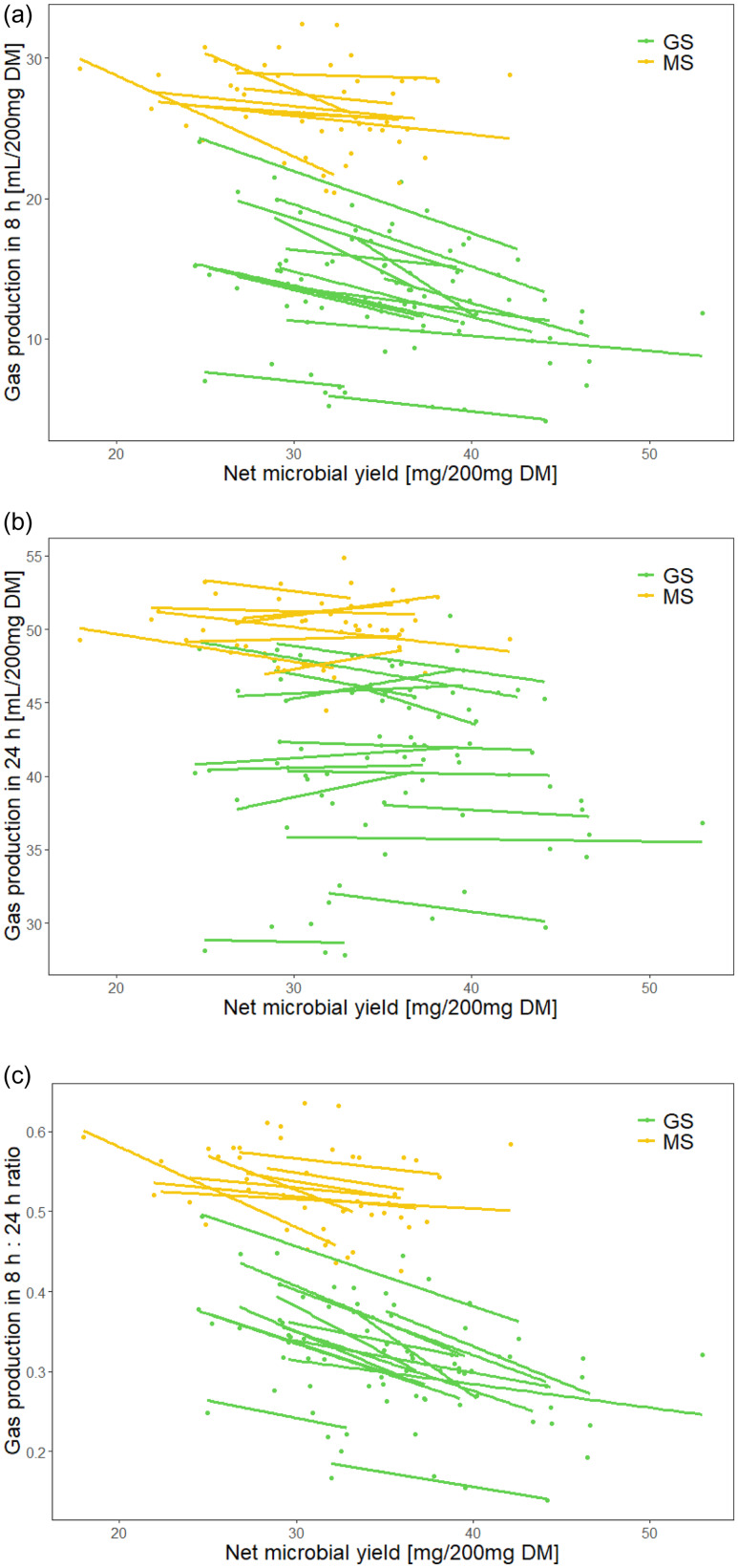
Comparison between net microbial yield and gas production in 8 h and 24 h and the ratio between gas production in 8 h and 24 h. The linear regression fitted line for each individual silage sample across six runs is given. GS, grass silage; MS, maize silage. The overall R^2^ for the linear mixed model with silage type and net microbial yield as fixed effects and silage individual as a random effect was (a) gas production in 8 h: R^2^_m_ = 0.75, R^2^_c_ = 0.91; (b) gas production in 24 h: R^2^_m_ = 0.44, R^2^_c_ = 0.97; and (c) ratio between gas production in 8 h and 24 h: R^2^_m_ = 0.75, R^2^_c_ = 0.88.

**Fig. 4.**
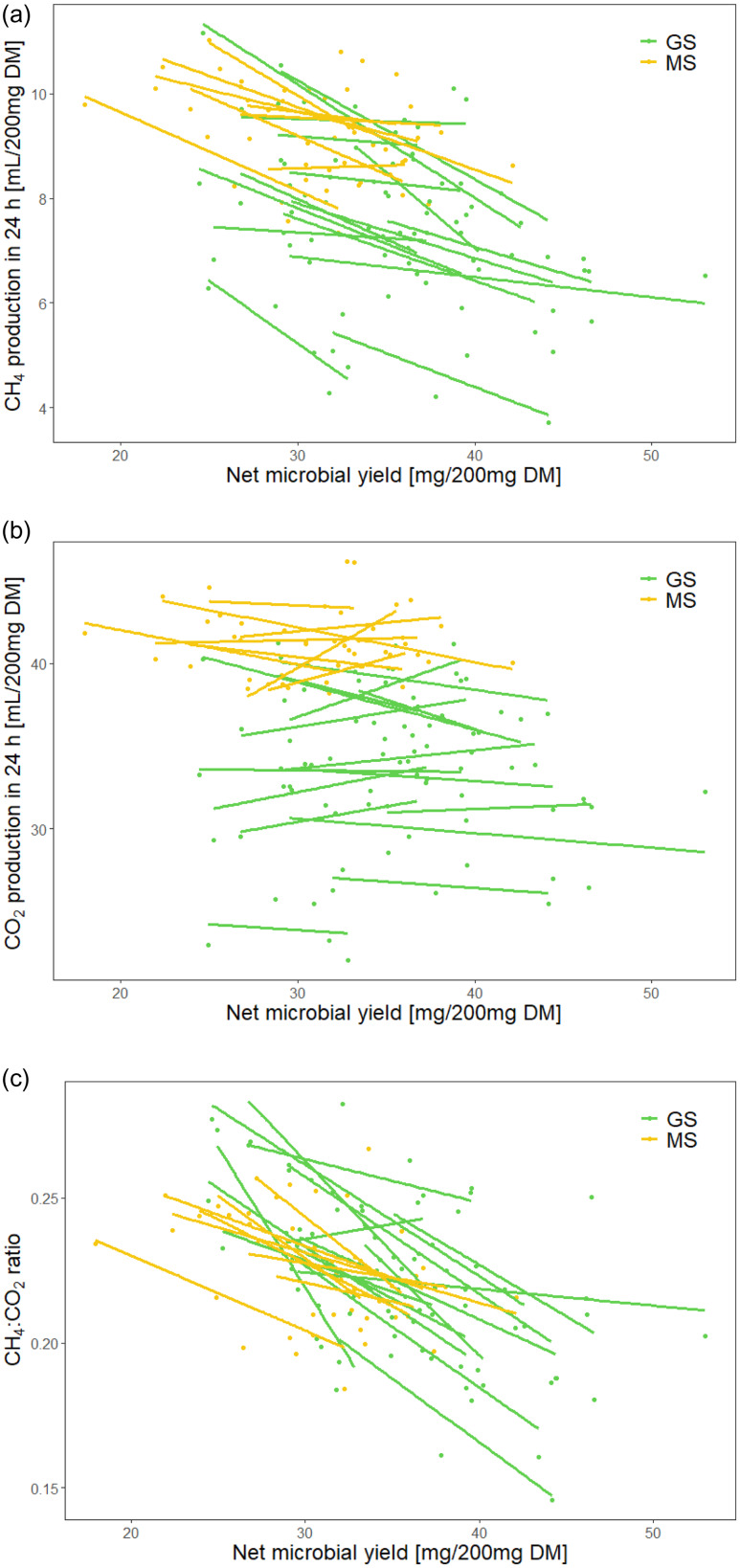
Comparison between net microbial yield and CH_4_ production, CO_2_ production, and the ratio between CH_4_ and CO_2_ production in 24 h. The linear regression fitted line for each individual silage sample across six runs is given. GS, grass silage; MS: maize silage. The overall R^2^ for the linear mixed model with silage type and net microbial yield as fixed effects and silage individual as a random effect was (a) CH_4_ production in 24 h: R^2^_m_ = 0.37, R^2^_c_ = 0.82; (b) CO_2_ production in 24 h: R^2^_m_ = 0.45, R^2^_c_ = 0.91; and (c) ratio between CH_4_ production and CO_2_ production: R^2^_m_ = 0.30, R^2^_c_ = 0.56.

In all relationships examined via linear mixed models, a significant effect was identified for silage type as a categorical variable (P<0•050), which indicates a notable difference between grass and maize silage within the context of these relationships (with the exception when the CH_4_:CO_2_ ratio acted as the dependent variable). A strong negative relationship was detected for gas production after 8 h and the net microbial yield (P < 0•001; R^2^_m_ = 0.75, R^2^_c_ = 0.91), but not for gas production after 24 h (P = 0•052; R^2^_m_ = 0.44, R^2^_c_ = 0.97); a negative relationship was also detected between the net microbial yield and the ratio between gas production after 8 h and 24 h (P < 0•001; R^2^_m_ = 0.75, R^2^_c_ = 0.88) (Fig. 3). In other words, when more microbial mass was produced, gas production was particularly low in the initial phase of fermentation. For the gas produced up to 24 h, a negative relationship with net microbial yield was detected for CH_4_ (P < 0•001; R^2^_m_ = 0.37, R^2^_c_ = 0.82), but not for CO_2_ (P = 0•192; R^2^_m_ = 0.45, R^2^_c_ = 0.91); correspondingly, the ratio between CH_4_ and CO_2_ production correlated negatively with net microbial yield (P < 0•001; R^2^_m_ = 0.30, R^2^_c_ = 0.56) (Fig. 4).

**Fig. 5.**
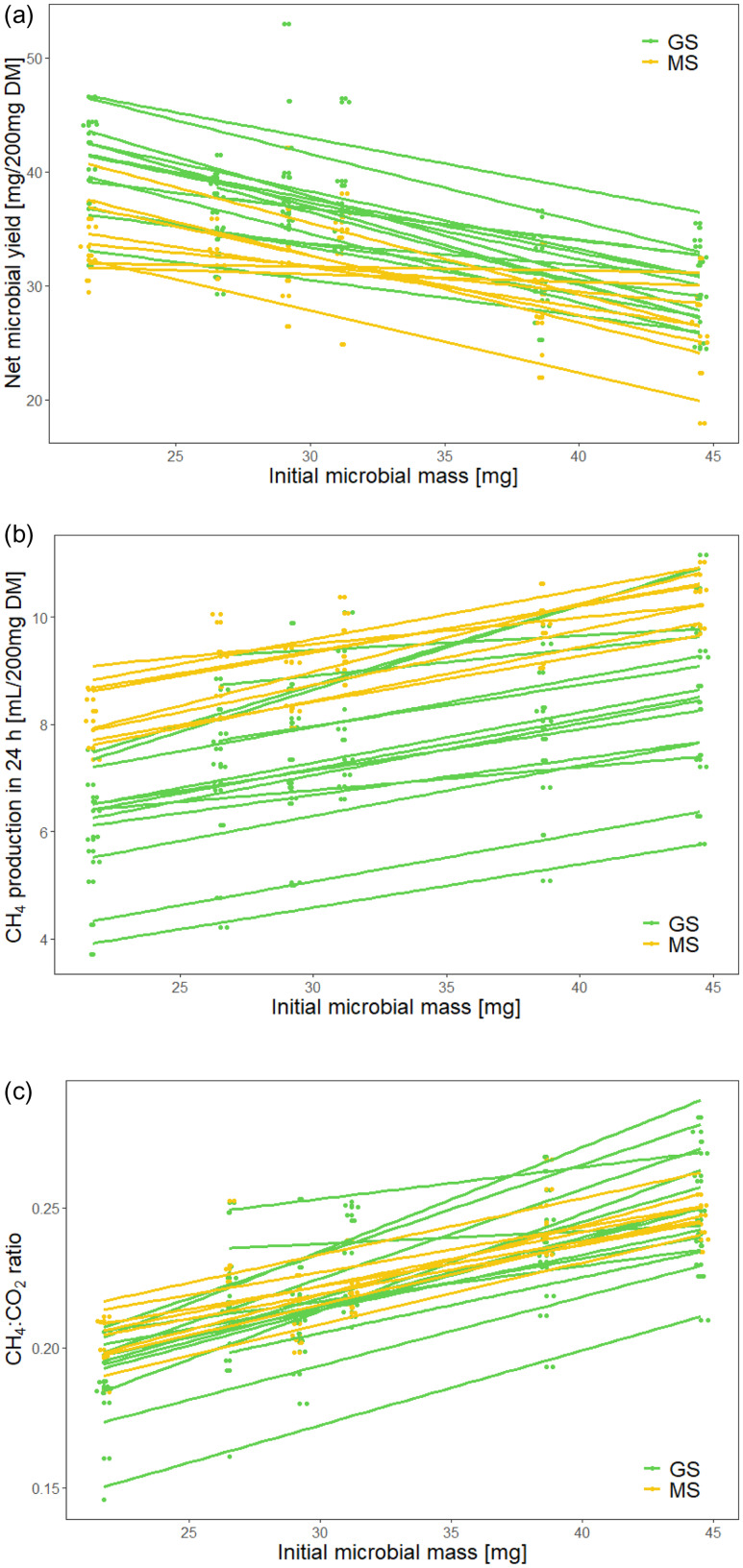
Comparison between initial microbial mass and net microbial yield, CH_4_ production, and the ratio between CH_4_ and CO_2_ production in 24 h. The linear regression fitted line for each individual silage sample across six runs is given. GS, grass silage; MS: maize silage. The overall R^2^ for the linear mixed model with silage type and initial microbial mass as fixed effects and silage individual as a random effect was (a) net microbial yield: R^2^_m_ = 0.46, R^2^_c_ = 0.61; (b) CH_4_ production in 24 h: R^2^_m_ = 0.45, R^2^_c_ = 0.91; and (c) ratio between CH_4_ production and CO_2_ production: R^2^_m_ = 0.46, R^2^_c_ = 0.76.

**Fig. 6.**
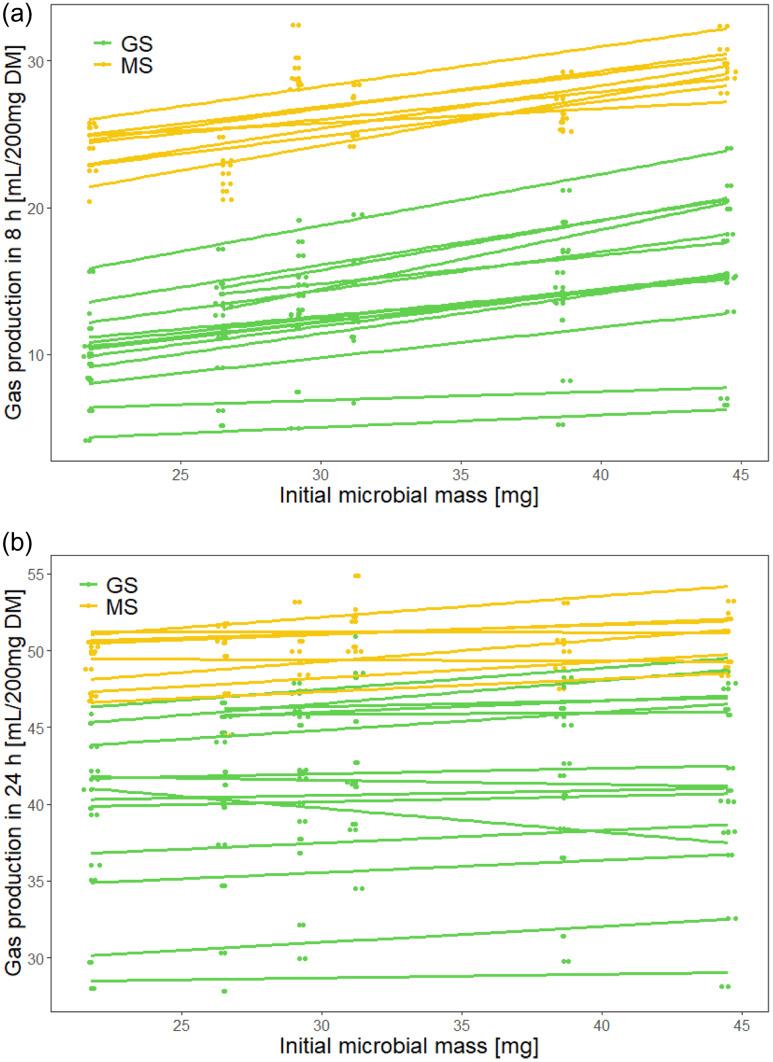
Comparison between initial microbial mass and gas production in 8 h and 24 h. The linear regression fitted line for each individual silage sample across six runs is given. GS, grass silage; MS, maize silage. The overall R^2^ for the linear mixed model with silage type and initial microbial mass as fixed effects and silage individual as a random effect was (a) gas production in 8 h: R^2^_m_ = 0.79, R^2^_c_ = 0.95 and (b) gas production in 24 h: R^2^_m_ = 0.45, R^2^_c_ = 0.97.

Initial inoculum microbial mass was negatively related to the net microbial yield (P < 0•001; R^2^_m_ = 0.46, R^2^_c_ = 0.61) (Fig. 5). In other words, net microbial yield was greater if the initial microbial mass was low. Consequently, a higher initial microbial mass lead to a higher CH_4_ yield (P < 0•001; R^2^_m_ = 0.45, R^2^_c_ = 0.91) and a higher CH_4_:CO_2_ ratio (P < 0•001; R^2^_m_ = 0.46, R^2^_c_ = 0.76) (Fig. 5). The initial inoculum microbial mass correlated positively with gas production after 8 h (P < 0•001; R^2^_m_ = 0.79, R^2^_c_ = 0.95) and after 24 h (P < 0•001; R^2^_m_ = 0.45, R^2^_c_ = 0.97) (Fig. 6).

## Discussion

This study modified the incubation experiment of Blümmel *et al.* ^(9)^ measuring degraded substrate, SCFA (acetate, propionate, butyrate), CH_4_, and microbial mass. While Blümmel *et al.* ^(9)^ only incubated straws, our study also included maize silage; according to our knowledge, the starch content in maize silage is typically fully degraded after 24 h.^(16,17)^ It must be noted that microbial mass measurements were only taken at 24 h, not at 8 h; therefore, the measurements used in this study should not lead to an overestimation of microbial mass for maize silages due to the starch content. As a batch culture system, HGT offers advantages for studying fermentation balance and electron flow, facilitating complete and accurate collection of fermentation end products to investigate fermentation direction and electron flow under controlled experimental conditions. In doing so, it is important to base calculations concerning microbial mass on OM instead of DM, to standardise values by the amount of incubated substrate, and to focus on net production instead of static concentration values that do not differentiate between the baseline introduced by the inoculum and the change during fermentation.

**Fig. 7.**
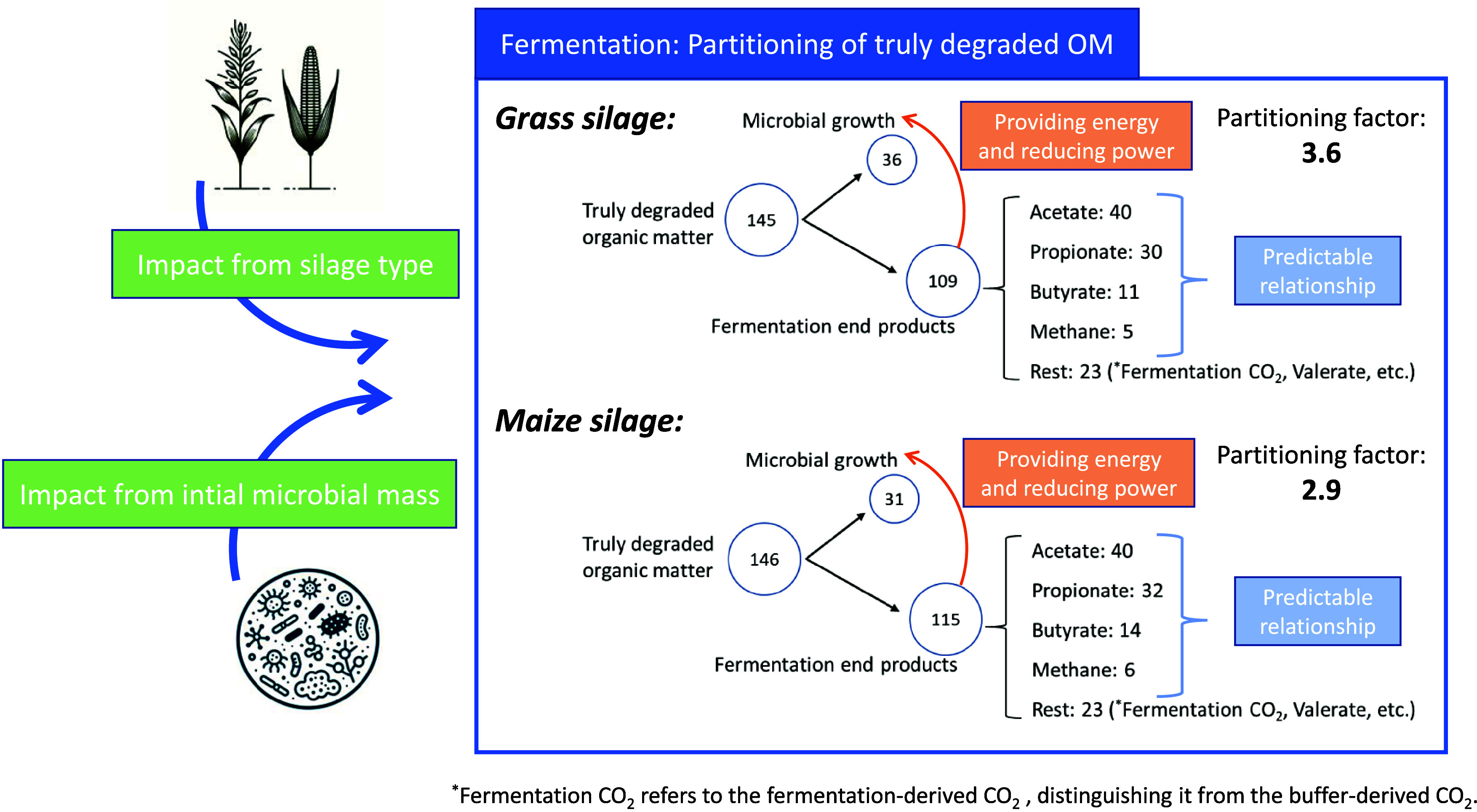
Schematic of effect of silage type and initial microbial mass on fermentation partitioning. The partitioning of truly degraded OM is presented as net production in mass (mg per 200 mg DM of incubated substrate) during 24 h of fermentation, calculated as the product of the substance’s amount and its molar mass. The images of grass, maize, and microbes on the left were generated by ChatGPT version GPT-4.

The higher CH_4_ production by maize silages is suggested to be led by higher total gas production (because the ratio between CH_4_ and CO_2_ was similar). Thus, for grass silages, less degraded substrate was directed to gas production but more to microbial synthesis, which is also indicated by a higher partitioning factor (Fig. 7). Higher microbial synthesis by grass than maize silage was also reported by several other studies^(18–20)^; for example, the *in vitro* trial by Boguhn *et al.* ^(20)^ incubating grass and maize silage reported similar results, with higher protein and fibre content for grass silage leading to higher OM degradability and microbial yield compared to maize silage, although they reported no difference in gas production. The higher crude protein content in grass silages might promote the proliferation of microbial biomass. Therefore, a higher microbial yield might also be related to a higher OM degradability for grass silage. Another cause might be differences in the polysaccharide structure between cellulose and starch. The transformation from polysaccharide to monosaccharide is mainly accomplished by hydrolysis, which is a non-redox reaction; however, the complex structure of plant cell wall might postpone the principal metabolic pathways and also select the microbial population. Although not measured in our experiment, differences in the microbial population after the fermentation of grass and maize silages are expected,^(21,22)^ which is assumed to be responsible for the different fermentation patterns.

The stoichiometric relationship between SCFA and CH_4_ has been suggested by several studies,^(1,3,6)^ though few studies have verified this experimentally. Robinson *et al.* ^(23)^ concluded that *in vivo* SCFA concentrations could hardly serve as an indicator to predict CH_4_ production, probably because of the difficulty in measuring the net production of fermentation end products, of which the SCFA is absorbed through the rumen wall. By contrast, in a batch culture system, the produced fermentation end products accumulate and can be measured relatively precisely. Theoretically, the 2H recovery should be 100% in a system (such as the rumen or HGT incubation system); this is because all produced reducing power must be utilised (according to the principle of redox conservation). Our results of 2H recovery (only considering SCFA and CH_4_) support our assumption that the main 2H recovery is accomplished by the production of SCFA (acetate, propionate, and butyrate) and CH_4_. Discrepancies exist with other studies concerning the calculated 2H recovery, and a lower or much lower than a 100% recovery is reported in batch culture^(5,24)^ and continuous culture.^(5,25)^ Our results showed that the initial microbial mass, as well as SCFA concentration, can constitute more than half of the end-stage. For example, Ungerfeld *et al.* ^(24)^ minimised the effect of the initial values through dilution of the inoculum from 20% to 0.1% — but this might influence the fermentation pattern values. With respect to our findings, a dilution of the inoculum (i.e. a reduction of microbe mass) might lead to a fermentation process directed more towards microbial growth and less towards gas production. This could represent a promising area of future research.

Microbial synthesis pathways like fatty acids elongation and NH_3_ incorporation consume 2H; many *in vitro* and *in vivo* studies indicated that increasing microbial synthesis helps redirecting 2H flow and, therefore, reduces CH_4_ production.^(8,26–28)^ The negative relationship between microbial and CH_4_ production was also confirmed by our results. Demeyer *et al.* ^(10)^ estimated that the synthesis of 1 kg microbial cells consumed 6.1 mol 2H; later work by Baldwin *et al.* ^(29)^ and Benchaar *et al.* ^(11)^ reported the hydrogen requirement of microbial growth without preformed amino acids is 2.71 mol 2H per kg microbes, while Mills *et al.* ^(12)^ reported 0.41 mol 2H per kg microbes. The estimation range of 0.41–6.1 mol is due to varying methodologies utilising stoichiometric relationships based on assumptions, such as Demeyer’s assumption regarding the chemical composition of ruminal microbes, which is suggested to be not constant. If we take the estimation of Demeyer *et al.*,^(10)^ then the amount of 2H consumed by microbial synthesis accounted for 19.7% and 13.9% of the 2H consumption by CH_4_ production for grass and maize silages, respectively. However, it is not possible to verify this through our experiment, since we obtained a higher than 100% 2H recovery. Thus, other 2H production pathways likely exist beyond acetate and butyrate generation. For example, Baldwin *et al.* ^(29)^ and Benchaar *et al.* ^(11)^ estimated that microbial growth with preformed amino acids produced 0.42 mol 2H per kg microbes; although this value of 2H production is substantially lower than their estimation of 2H consumption, the extent to which it affects electron flow remains a subject for further discussion.

Under the situation of limiting amounts of available nutrients, the lower initial microbial mass might direct more energy and nutrients towards microbial growth and thus attain a higher growth rate, as indicated by our results. A higher microbial gain indicates more truly degraded substrate was directed to microbial synthesis and less to SCFA, resulting in a higher PF, and because 2H is consumed in the process, there is less CH_4_ production (as shown in Fig. 7, for grass silage, higher microbial yield might direct truly degraded OM more from fermentation end products to microbial growth, resulting in less CH_4_ production). When assessing the difference between measured gas production and the predicted gas production based on SCFA release during NDF digestion *in vitro*, Doane *et al.* ^(30)^ found that this difference was only partially explained by the amount of propionate released and suggested that microbial synthesis might be responsible for the remaining discrepancy. In their study, this discrepancy was mainly observed in the initial stages of *in vitro* fermentation, similar to our findings that initial microbial mass and gas production are negatively related at the initial 8 h gas reading. Assuming that *in vitro* microbial biomass production will be constrained at some point by the limited habitat available in the *in vitro* system used, as neither microbes nor gases are removed during the assay, this may be particularly relevant *in vivo* where microbes and fermentation gases are removed constantly, and conditions in the reticulorumen might therefore be most represented by assuming conditions of the initial *in vitro* fermentation for extended time periods.

Although there are already studies concerning the fermentation kinetics of total gas production^(31–35)^ and fermentation end products as well as microbiomes,^(30,36)^ there is a need to conduct more kinetic studies with the complete collection of fermentation end products and various microbial types and incorporating transcriptomics to understand the metabolic state of the microbiome at different growth stages. Additionally, it should be further explored how the fermentation direction and electron flow are influenced not only by substrate but also by the inoculum’s initial microbial mass or by the constant removal of microbes from open systems.

## Supporting information

Zhang et al. supplementary material Zhang et al. supplementary material

## Supplementary material

The supplementary material for this article can be found at https://doi.org/10.1017/jns.2024.37.
