# Supplementary material for: Inoculum microbial mass is negatively related to microbial yield and positively to methane yield in vitro
Source: J Nutr Sci. 2024 Sep 20;13:e44. doi: 10.1017/jns.2024.37 (PMC11428107; doi:10.1017/jns.2024.37)
Supplement: Zhang et al. supplementary material [file S2048679024000375sup001.docx]

**Appendix** Table of established linear regression between true OM degradability as an independent variable and short chain fatty acid (SCFA) concentration as a dependent variable for grass silage samples in six rounds, for estimation of initial SCFA concentration in the inoculum, which was assumed to be the intercept.

| Round | Slope | | | Intercept | | | R^2^ | | |
| --- | --- | --- | --- | --- | --- | --- | --- | --- | --- |
|  | Acetate | Propionate | Butyrate | Acetate | Propionate | Butyrate | Acetate | Propionate | Butyrate |
| 1 | 0.23 | 0.17 | 0.02 | 22.31 | 2.15 | 3.57 | 0.55 | 0.28 | 0.23 |
| 2 | 0.26 | 0.17 | 0.05 | 22.73 | 1.45 | 1.75 | 0.56 | 0.45 | 0.45 |
| 3 | 0.31 | 0.18 | 0.06 | 19.20 | 0.32 | 1.60 | 0.78 | 0.55 | 0.59 |
| 4 | 0.26 | 0.13 | 0.06 | 28.32 | 5.20 | 2.63 | 0.56 | 0.42 | 0.51 |
| 5 | 0.28 | 0.18 | 0.05 | 22.57 | 0.13 | 2.03 | 0.54 | 0.48 | 0.46 |
| 6 | 0.31 | 0.16 | 0.08 | 19.79 | 1.56 | 0.00 | 0.70 | 0.48 | 0.06 |
